# Supplementary material for: Pain Education in the Wellness, Training Performance, and Pain Intensity of Youth Athletes: An Experimental Study
Source: Healthcare (Basel). 2024 Jan 16;12(2):215. doi: 10.3390/healthcare12020215 (PMC10815667; doi:10.3390/healthcare12020215)
Supplement: Supplementary file 1 [file healthcare-12-00215-s001.zip › Supplementary tables.pdf]

Table S1 Wellness and pain intensity of female athletes

	Control Group				Intervention Group				Between-group differences	
	PRE Mean (SD)	POST Mean (SD)	Intra-group differences		PRE Mean (SD)	POST Mean (SD)	Intra-group differences			
			Mean (95%CI)	ES			Mean (95%CI)	ES	Mean (95%CI)	ES
Pain intensity	3.00 (1.00)	4.50 (1.80)	-1.50 (-7.95; 4.95)	NS	-	-	-	-	1.57 (-0.08; 3.22)	NS
Sleep	3.64 (1.59)	3.28 (1.49)	0.36 (-0.89; 1.61)	NS	3.15 (1.62)	3.84 (1.34)	-0.69 (-1.48; 0.10)	NS	-0.56 (-1.69; 0.56)	NS
Stress	3.50 (1.51)	3.92 (1.21)	-0.43 (-1.06; 0.20)	NS	4.69 (1.84)	3.77 (1.36)	0.92 (0.17; 1.82)*	0.46	0.16 (-0.86; 1.18)	NS
Fatigue	4.14 (1.17)	3.85 (1.35)	0.28 (-0.24; 0.81)	NS	4.38 (0.77)	4.31 (0.85)	0.07 (-0.64; 0.79)	NS	-0.45 (-1.35; 0.45)	NS
Muscle soreness	3.92 (1.33)	4.28 (1.43)	-0.35 (-1.54; 0.83)	NS	4.00 (1.15)	3.46 (1.33)	0.53 (-0.53; 1.60)	NS	0.82 (0.53; -0.27)	NS
Wellness	15.21 (3.55)	15.35 (3.20)	-0.14 (-2.08; 1.80)	NS	16.23 (3.03)	15.38 (2.98)	0.84 (-0.43; 2.12)	NS	-0.27 (-2.48; 2.42)	NS

CI, confidence interval; ES, effect size; NS, non-significant; SD, standard deviation; *$p < 0.05$

Table S2 Wellness and pain intensity of male athletes.

	Control Group				Intervention Group				Between-group differences	
	PRE Mean (SD)	POST Mean (SD)	Intra-group differences		PRE Mean (SD)	POST Mean (SD)	Intra-group differences			
			Mean (95%CI)	ES			Mean (95%CI)	ES	Mean (95%CI)	ES
Pain intensity	4.00 (1.41)	7.50 (0.70)	-3.50 (-22.56; 15.56)	NS	6.00 (1.41)	4.50 (0.70)	1.50 (-4.85; 7.85)	NS	1.40 (-1.79; 4.59)	NS
Sleep	3.50 (1.00)	3.66 (1.23)	-0.17 (-0.69; 0.36)	NS	3.33 (1.50)	3.44 (1.13)	-0.11 (-1.47; 1.24)	NS	0.22 (-0.87; 1.32)	NS
Stress	3.91 (1.67)	3.33 (1.37)	0.58 (-0.33; 1.50)	NS	3.78 (0.97)	3.22 (1.48)	0.55 (-0.78; 1.89)	NS	0.11 (-1.19; 1.42)	NS
Fatigue	4.83 (1.40)	4.25 (0.86)	0.58 (-0.33; 1.50)	NS	4.22 (1.78)	4.11 (1.16)	0.11 (-1.18; 1.14)	NS	0.14 (-0.79; 1.06)	NS
Muscle soreness	3.58 (1.67)	4.17 (1.27)	-0.58 (-1.41; 0.25)	NS	4.00 (1.93)	4.11 (1.27)	-0.11 (-1.93; 1.71)	NS	0.55 (-1.11; 1.22)	NS
Wellness	15.83 (3.66)	15.41 (3.03)	0.41 (-1.43; 2.26)	NS	15.33 (2.87)	14.89 (3.48)	0.44 (-3.12; 4.01)	NS	0.52 (-2.55; 3.61)	NS

CI, confidence interval; ES, effect size; NS, non-significant; SD, standard deviation; *$p < 0.05$
