# Supplementary figures and images for: Pain Education in the Wellness, Training Performance, and Pain Intensity of Youth Athletes: An Experimental Study
Source: Healthcare (Basel). 2024 Jan 16;12(2):215. doi: 10.3390/healthcare12020215 (PMC10815667; doi:10.3390/healthcare12020215)

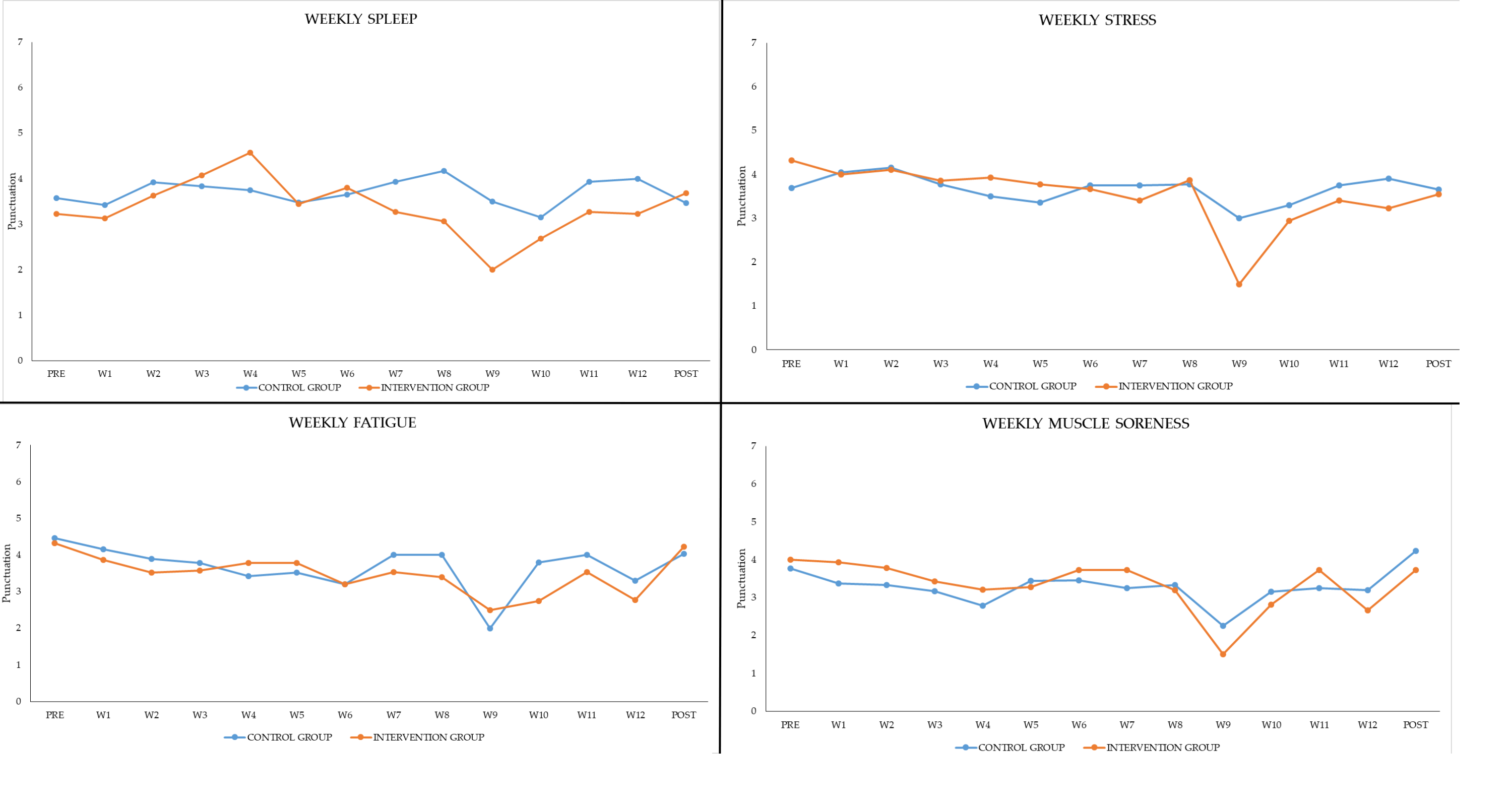

Supplement: Supplementary file 1 [file healthcare-12-00215-s001.zip › Figure S1. Wellness week.png]
